# Examining the Risks and Difficulties Related to Noncompleted and Completed Sexual Offences Against Adult Women: Insights on Decision Making for Crime Prevention

**DOI:** 10.1177/10790632261470420

**Published:** 2026-07-21

**Authors:** Benoit Leclerc, Richard Wortley, Danielle Reynald, Jesse Cale

**Affiliations:** 1 School of Criminology and Criminal Justice, 5723 Griffith University, Brisbane, QLD, Australia; 2 Te Puna Haumaru New Zealand Centre for Security and Crime Science, Waikato University, Hamilton, New Zealand; 3 Arts, Education and Law, 5723 Griffith University, Brisbane, QLD, Australia

**Keywords:** sexual offences, sexual crime, sexual offending, rape, guardianship, self-protection, CPTED, situational crime prevention, risk of getting caught, offender-based research

## Abstract

Using a sample of 145 men convicted for sexually assaulting a woman, we examined perceived risks and difficulties in the context of noncompleted and completed sexual offences against women to help further understand the decision-making process of individuals who commit those crimes and offer complementary insights from a prevention point of view. The participants were 29 years old on average. We looked at the individual’s accounts of their offending. First and foremost, the dominating response in regards to high ratings of difficulties in noncompleted offences was woman resistance. Second, intoxication was reported by participants to justify both high risk and the absence of risk during their offending. Third, participants who perceived no risk and completed a sexual offence against a woman further indicated, in almost half the cases, that they thought (or assumed) the woman had given her consent initially. Fourth, very few participants referred to environmental conditions when assessing the risks and difficulties of committing an offence. Lastly, the results are discussed in light of considerations for crime prevention.

## Introduction

Crime prevention initiatives that increase the perceived risks and effort associated with offending are embedded in everyday life, often without people realising it. Examples include CCTV surveillance, improved lighting, security personnel, and electronic tagging of retail goods. While most individuals pay little attention to these measures, they are particularly salient to those who might otherwise be tempted to offend. Potential individuals who commit crimes will take into account the risk of getting caught and the difficulties related to crime commission when considering crime and/or what might happen if they initiate a crime (e.g., Cornish & Clarke, 1986). In addition, the type of crime one commits will arguably involve different risks and difficulties, which generally correlate with the degree of seriousness of the crime. How those who commit crime assess those risks and difficulties to commit sexual offences is generally not well documented, which limits insights that can inform prevention initiatives for reducing crime through evidence-based research.

In this study, we seek to contribute to the small body of knowledge on the decision-making process of individuals who commit sexual offences against women (e.g., Beauregard et al., 2012; Beauregard & Leclerc, 2007). What works and what does not in terms of prevention measures is often not well documented through empirical evidence simply because of the difficulty of evaluating any program that is meant to prevent a sexual offence (Wortley & Smallbone, 2006). To help gain further understanding of the decision-making processes of individuals who commit sexual offences against women, two interrelated questions are examined: (1) What are the factors considered by individuals that led them to determine that the situation *did not represent sufficiently high risk or difficulties* that would stop them from offending during completed sexual offences against women? (2) What are the factors considered by individuals that led them to determine that the situation *involved sufficiently high risks of getting caught and difficulties* to discontinue the offence resulting in noncompleted sexual offences against women? One particular novelty in this study is the opportunity to examine the decision-making process of individuals in the context of noncompleted sexual offences.

## Theoretical Framework

In criminology, the idea of a calculated individual who makes an assessment of the cost and benefit before committing crime comes from the rational choice perspective (Cornish & Clarke, 1986). In their assessment, those individuals are likely to consider the risk of getting caught as well as the difficulties related to committing a crime, whether those difficulties involve neutralising an alarm, getting access to a property or target without being seen, overcoming the resistance of a victim, or obtaining specific tools to commit crime. The tenets of rational choice indicate that this cost and benefit assessment is less than optimal as the decisions are likely to be affected by missing information, intoxication, and emotions that may affect their ability to make a sound decision (e.g., Clarke, 2014; Cornish & Clarke, 1987). This principle has been applied to sexual crimes previously (Cornish & Clarke, 2008). For instance, in a study of 553 adult males who had been convicted for sexual offences in Canada, Leclerc and Lindegaard (2018) demonstrated that the emotions of those committing sexual offences fluctuated across the stages of the crime event, with these emotional transitions influencing how they think and act during the offence (see also van Gelder et al., 2014). Indeed, emotions in decision making can have a substantial impact on whether or not an individual decides to commit an offence in the first place, or whether or not to discontinue an offence when facing resistance by a victim or encountering a bystander (see van Gelder, 2018; van Gelder et al., 2014).

The rational choice perspective originated from the need to situate situational crime prevention (SCP) into a theoretical approach in criminology (Cornish & Clarke, 1986). At the core of SCP is the idea that the cost-benefit analysis performed by those who commit crimes can be altered by environmental changes to reduce criminal opportunities (Newman et al., 1997). Taken broadly, SCP seeks to alter situations to make them more risky and less rewarding and thus less attractive to individuals who might be likely to make the decision to offend. At its foundation, and consistent with the rational choice perspective, the SCP approach incorporates the person-situation interaction as a premise into the theoretical foundation and the development of the classification of situational prevention techniques (see Clarke & Eck, 2003), which is essential to understand the dynamic of crime commission (e.g., Leclerc et al., 2009; Leclerc et al., 2016; Wortley, 2012). It is reasonable to think that without a motivated individual, crime should not occur. Likewise, without an opportunity to offend, crime is less likely. Logically speaking, the less criminal opportunities there are, the less likely to observe crime (Clarke, 2008). The rational choices about what to do in a situation will also change if the situation changes, making a compelling case for the critical importance of the situation in criminology (Wortley & Tilley, 2018).

The classification of SCP techniques originated with three techniques focused on reducing offending opportunities, namely increasing risks and efforts for individuals to commit crime and reducing rewards obtained through crime (Clarke, 1997). Following this development, Wortley (2001) argued that the application of SCP was limited to reducing criminal opportunities. As described by Wortley, the environment can also affect the psychological readiness of an individual to offend. The individual will select cues from the environment that will reduce moral inhibitions or increase the sense of excitement or thrill, for instance. Based on this theoretical development to SCP, Homel and Clarke (1997) revised the classification techniques framework and added two strategies focused on the individuals who commit crimes, that is, removing excuses and reducing provocations that could precipitate individuals to offend. The current SCP framework comprises the following techniques: (1) Increasing risks, (2) Increasing effort, (3) Reducing rewards, (4) Removing excuses, and (5) Reducing provocations (see also Cornish & Clarke, 2003).

## Previous Literature on Sexual Offences Against Women

Empirical literature on the assessment of risks and difficulties for those who commit sexual offences against women is very scarce. Beauregard and his colleagues (2007; 2012) examined the responses of individuals who committed sexual offences to understand their rationales during the crime event. Semi-structured interviews were conducted with a sample of 69 individuals who committed serial sex crimes against stranger victims in order to identify the rationale behind their actions during the pre-crime phase (premeditation of the crime, estimation of risk of getting caught, and forensic awareness), crime phase (use of a weapon, restraints, vehicle, and physical force), and the post-crime phase (event leading to the end of crime and victim release site location choice). Results showed that these individuals considered many factors in their decision to offend, which include the risk of getting caught. Almost a third indicated that they never thought about the risk of getting caught (e.g., “You don’t think about that, you don’t have the time, it just happens and it’s after, when you cool down at this stuff comes up”) (p. 121). Around four in 10 of those who considered high risk of getting caught reported that this was due to the presence of a guardian (bystander), the nature of the crime location (public or the victim’s home), and the fact that the victim had information about them or not. In addition, an equal proportion (39%) reported using a weapon. In these cases, overcoming victim resistance was the main reason provided by participants (85%). Of those who used restraints (14%), 30% indicated doing so to prevent resistance. Of those who did not use physical force, the absence of victim resistance was reported as the main reason for not doing so (63%). Finally, of those who used instrumental physical force (35%), most reported doing so to control the victim (85%). Victim resistance (71%) was reported as the main justification for using more physical force than necessary by those who did so (35%).

Other empirical studies have added complementary findings to studies by Beauregard and his colleagues (2007; 2012). In a study of 366 adult males incarcerated for a contact sexual offence, Leclerc et al. (2022) collected self-report data on participants’ offending behaviour. Findings from this study indicated that individuals who attempted to commit a sexual offence against women but were discouraged from doing so, rated the difficulties related to crime commission to be high, an assessment that was correlated with the level of victim resistance. Through the use of hypothetical scenarios with participants, another study looked at the perceived effectiveness of self-protection, crime prevention through environmental design measures (CPTED), and guardianship to prevent sexual offences against women (Chiu et al., 2021). The three hypothetical scenarios involved potential sexual offences against women. Those scenarios described an incident where a man wished to proceed with sexual contact but the woman did not. Participants were then asked to rate the perceived effectiveness of a range of potential SCP measures relating to three strategies: guardianship (potential arrival or nearby presence of a third party), victim self-protective behaviour (victim related factors), and environmental design (security measures embedded in the environment). Where offences occurred in the individual’s home with a known victim, victim self-protection was seen as the most effective measure. In these situations, more forceful forms of self-protection were perceived to be effective, including yelling for help, threatening to report the incident to the police, and running away. In this context, one could reasonably expect those individuals to perceive a high risk of getting caught when the woman is using those self-protective measures. Where the offense occurred in a public location with a stranger victim, although the perceived effectiveness of victim self-protective behaviour remained constant, guardianship and environmental design were seen as significantly more effective.

Building on this research stream (see Chiu et al., 2021; Cook et al., 2018; Cook et al., 2020; Leclerc et al., 2022; 2023), Chopin et al. (2024) recently conducted a study to examine the factors that could determine why a sexual offence is disrupted with documented cases from the police in France. Consistent with Leclerc and his colleagues, the authors defined noncompletion “as interruption or event that curtailed the offense took place either before or during the commission of sexual acts” (p. 4). For instance, findings indicated that cases occurring during daylight involving younger victims were more likely to be interrupted by a third party. Another finding indicated that when an individual met and attempted a sexual assault at the same location, victims were more likely to escape. Victim resistance also appeared as an important variable to understand the disruption of the offence. Finally, consistent with Leclerc and his colleagues’ study, the authors found different patterns of interrupted offences between child and women victims of sexual offence. It is relevant noting that the relationship between perceived risks and difficulties and factors that can shape disruption were not examined in that study.

## Current Study

Individuals who commit sexual offences are often viewed as weighing the cost and benefit of criminal opportunities prior to offending. Although this assessment is often imperfect and influenced by factors such as intoxication, it can nevertheless be argued that individuals who commit crime are still engaging in a form of decision-making when doing so (Clarke, 2014). Investigating this aspect of decision-making necessitates first-hand data collected from those individuals. To our knowledge, only a few studies have looked at decision-making of individuals who committed sexual crimes in-depth (e.g., Beauregard et al., 2012; Beauregard & Leclerc, 2007). In addition, these studies used a specific sample of serial sexual crimes, which findings may be unrepresentative of most individuals committing sexual offences. Finally, individuals who commit crime can provide research with very rich insights into their decision-making process and what elements or circumstances of a situation they perceive to make the perpetration of an offence riskier and/or more difficult to carry out (Chiu et al., 2021; Leclerc et al., 2022). This study extends this body of work by highlighting its more in-depth qualitative focus on decision-making.

For prevention initiatives, and to better understand what works and what does not to prevent sexual offences against women, it is important to pursue this research agenda to better understand how individuals committing sexual offences are assessing situations to be risky and difficult to commit crime. To help us gain a better understanding of the decision-making process of individuals who commit sexual offences against women, two questions are examined:

Research question 1: For completed sexual offences against women, what are the factors considered by individuals that led them to determine that the situation *did not involve sufficiently high risks of getting caught or difficulties* that would stop them from offending?

Research question 2: For noncompleted sexual offences against women, what are the factors considered by individuals that led them to determine that the situation *involved sufficiently high risks of getting caught or difficulties* to discontinue the offence?

## Methodology

### Sample

A sample of 363 adult males convicted of sexual offences against a woman or child incarcerated in Queensland, Victoria, or Northern Territory (Australia) initially participated in a funded research project on the situational prevention of sexual offences. As the focus of this study was on sexual offences committed against women specifically, only adult males serving a custodial sentence for one or more sexual offences against a woman were selected for the current study (*n* = 145) (218 individuals were convicted for a child sexual offence and thus not included). Participants were on average 29 years old at their most recent sexual offense (*SD* = 12.9). Victims were on average 28.41 years old (*SD* = 10.14). A total of 59.4% of participants reported an Aboriginal/Torres Strait Islander ethnic background, 31.5% indicated not being in a romantic relationship, and 67.8% reported not completing Secondary school (2 missing data). Finally, 65.7% indicated currently or previously participating in a treatment program with Corrective Services (2 missing data). A minority of participants also indicated a previous history for sexually offending against a woman (14.1%) and/or a child (7.7%) (3 missing data).

### Data Analysis

Participants were asked to complete a comprehensive self-report questionnaire about their offending behaviour and decision-making process. Of 145 participants who were incarcerated for committing a sexual offence against a woman, 67 identified an incident in which they were stopped or discouraged either before or during the incident. The remaining 78 participants sexually assaulted a woman without being stopped. For the purpose of this study, only two variables of interest were selected from the questionnaire for analysis: (1) risks of getting caught and, (2) difficulties related to committing the offence. Risks of getting caught specifically refers to the individual’s assessment that the situation involves risks of getting caught if he pursues with committing an offence and, in this context, represents a broader assessment of the situation. Difficulties related to committing an offence refers to any effort or obstacle that the individual perceives as necessary to overcome to commit the offence, which may or may not be perceived as representing (or contributing to) a risky situation. These variables were collected as follows. Two sections of the questionnaire were relevant to the current study. The first section comprised questions about the most recent time participants tried to sexually victimised a woman but did not because they were stopped or discouraged either beforehand or during the incident. The second section comprised questions about the most recent time participants were caught for sexually victimising a woman. In each section (i.e., noncompleted and completed offences), participants were asked: (1) how they had perceived the risks of getting caught, and (2) the difficulties related to committing the crime. For each question, participants were asked to circle an answer ranging from 0–10, that is, from not risky at all (or not difficult) (i.e., score of 0) to very risky (or very difficult) (i.e., score of 10). Participants were also provided with the opportunity to explain why they had rated the risks and difficulties the way they did through an open-ended question. For both completed and noncompleted offences, these qualitative responses are examined in this study.

Although qualitative data lack numeric and statistical precision, they possess their own unique strengths. Insights on thought processes obtained through open-ended questions lend themselves to the discovery and analysis of unexplored paths (Jacques & Wright, 2008, 2011). In other words, the value of qualitative data resides in their ability to help build and illustrate new lines of research and theory (see Jacques & Wright, 2011; Laub & Sampson, 2003). Data were analysed manually and independent double coding was followed. Responses to open-ended questions were first classified under categories following a situational crime prevention framework by the first author (e.g., intoxication, women resistance). The last author then proceeded with the classification of the responses under the pre-determined categories to ensure the reliability of these categories and the responses representing them. Initially, for the noncompleted offences, 82.6% and 91.3% agreement was found for responses obtained for risks and difficulties, respectively. For the completed offences, 95.2% and 86.8% agreement was found for responses obtained for risks and difficulties, respectively. The first and last author then discussed the coding decisions and to resolve differences in discrepancies (O’Connor & Joffe, 2020). The purpose of these data is to illustrate how individuals who commit sexual offences are perceiving risks and difficulties related to committing crime, not to validate theory.

### Procedure

The corrective services departments in each of the three jurisdictions (Queensland, Victoria, and Northern Territory) identified convicted individuals in their facilities who were currently serving or had previously served a sentence for a sexual offence against a woman. Staff asked potential participants whether they would agree to learn about a research project on the prevention of sexual offences, and thereafter, if they agreed to participate. Participants who were involved in a therapeutic program were approached during one of their weekly group sessions, whereas individuals not participating in a program were approached individually to maintain their confidentiality. Participants who consented to participate completed the self-report questionnaire in the presence of at least one member of the research teams. This provided participants with the opportunity to ask any questions regarding the completion of the questionnaire. Once the questionnaire was completed, the research assistant checked the responses to make sure nothing had been misinterpreted or omitted. This assisted in minimizing missing fields in the data. Participants undergoing treatment completed the questionnaire individually in the group setting. Participants who were not undergoing treatment completed the questionnaire in a private room away from other prisoners. Prison authorities assigned each participant a unique identification number to be used in the research; to maintain privacy, the names of participants were not revealed to the researchers and no particulars were recorded that would allow an individual being identified. Ethical approval for this research was obtained from the Human Research Ethics Committee at Griffith University.

## Results

Descriptive statistics on the variables of interest are presented first. Perceived risks and difficulties related to the noncompleted sexual offence were originally scored between 0 (absence) to 10 (very high) by participants. Participants were asked to provide justifications for their rating. First, to examine the justifications provided by participants who did not perceive any risk or difficulties related to the commission of the offence, only justifications provided by participants who gave a score of 0 to risks or difficulties were considered (i.e., absence of any risk or difficulties). Second, to examine the justifications provided by participants for high risks and difficulties, we chose a cut-off score of 6 and above. Therefore, justifications for those who rated risks or difficulties between 6–10 were included below in the results. A score of 6 or higher arguably demonstrates that the participant perceived high risks or difficulties – simply because this score represents the threshold of an above-average score. This allowed us to identify the justifications most likely linked to perceived high risk or difficulties while retaining a variety of justifications to further understand the presence of risks and difficulties through the perspective of those who commit crimes (justifications for the ratings below 6 are available upon request).

Table 1 presents descriptive statistics for perceived risks and difficulties in noncompleted and completed sexual offences against women. Risks and difficulties were most often perceived as present by participants who were involved in a noncompleted offence compared to a completed offence (62.1% vs 46.2% and 75.8% vs 51.3%, respectively). Mean scores obtained on those variables were 4.05 vs 3.03 and 4.30 vs 2.33, respectively, for noncompleted offences compared to completed offences.

**Table 1.** Perceived Risks and Difficulties in Noncompleted and Completed Sexual Offences Against Women

Risks/difficulties	Noncompleted (*n* = 66)	Completed (*n* = 78)
Risks present (dichotomous % yes)	62.1% (*n* = 41)	46.2% (*n* = 36)
Risks rating 0-10 (continuous)^a^	4.05 (mean)	3.03 (mean)
	3.5 (median)	0 (median)
	3.9 (*SD*)	3.91 (*SD*)
Difficulties present (dichotomous % yes)	75.8% (*n* = 50)	51.3% (*n* = 40)
Difficulties rating 0-10 (continuous)^a^	4.30 (mean)	2.33 (mean)
	5 (median)	1.50 (median)
	3.41 (*SD*)	3.08 (*SD*)

^a^For the variables of risks and difficulties, data were collected using a Likert-type scale from 0 (absence of risks/difficulties) to 10 (very high risks/difficulties).

## Perceptions of Risks and Difficulties in Noncompleted Sexual Offences Against Women

Table 2 presents the justifications provided by participants who perceived high risks during a noncompleted sexual offence against a woman. The most common justifications provided by participants in determining the risk was too high were related to their intoxication (30.4%) and concerns of getting caught (21.8%). For many participants, being intoxicated represented a factor that may have increased the risk of getting caught by inhibiting their ability to think clearly. Consistent with this, many others reported concerns of getting caught. One participant said: “She was my girlfriend’s best friend and she told my girlfriend everything.” Some other participants reported that the woman was showing resistance (13%) and that there was a risk of a someone else seeing or hearing them where they initiated the offence (13%). Finally, some participants also reported other justifications (21.8%), such as losing control over their own behaviours, which again might relate to increased risk of getting caught.

**Table 2.** Justifications of Perceived High Risks in Noncompleted Sexual Offences Against Women (*n* = 23)

Justification for high risks	Frequency (%)
Intoxication	7 (30.4)
Very drunk and not thinking rationally	
Too drunk/stoned did it without thinking	
Drunk and angry	
Both us were drunk	
First time smoking cannabis and drinking at same time and wasn’t thinking straight	
Really drunk	
Was drunk and thought it was consensual	
Concerns of getting caught	5 (21.8)
She was my girlfriend’s best friend and she told my girlfriend everything	
Scared of getting caught	
Thought I might go to jail	
Because of getting caught	
Getting caught	
Woman showed resistance	3 (13)
She said No	
Stopped when she told me off	
When she turned me down, I got angry, snapped and adrenaline rushed and when she stopped struggling reality kicked in	
Environmental risk	3 (13)
Did not realise boyfriend was there	
Occurred in apartment so there were neighbours nearby	
Cars driving by	
Other	5 (21.8)
The excitement was the motivator	
I lost control of my behaviours	
The opportunity arose and I snapped	
It was the wrong choice because I was angry at my girlfriend and was taking it out on this woman	
Really wanted sex and didn’t want to kill her. Just sex	

Table 3 presents the justifications provided by participants in their own words who perceived high difficulties related to a noncompleted sexual offence against a woman. By far, the most common justification provided by participants was victim resistance (70%). One participant, for instance, said: “I was trying to talk her into it and force her but she was stopping me.” Another said: “She screamed and pushed me away. She was bigger than me.” In two additional cases, participants described both woman resistance and another contributing factor, either environmental risk or intoxication, as reasons for discontinuing the offence. Other reported justifications were their intoxication (9%), or some other isolated reasons, such as the failure to achieve arousal (13%).

**Table 3.** Justifications of Perceived High Difficulties in Noncompleted Sexual Offences Against Women (*n* = 23)

Justification for high difficulties	Frequency (%)
Woman showed resistance	16 (70)
Tried to kiss her. She fought back	
She used self-defence and it scared me off	
She screamed and pushed me away. She was bigger than me	
She did not want to have sexual contact	
She did not want me	
She was resisting	
I was trying to talk her into it and force her but she was stopping me	
She struggled at first but then conceded and said she would do it if I let her go	
She fought back	
She tried to fight back	
She was fighting back	
She was getting angry and telling me off	
She was resisting. I did not know what could happen.	
She would constantly refuse my advances till I forced her to allow me with a weapon	
The victim resisted - fought back also thought I would get caught	
Wife fighting back	
Intoxication	2 (9)
I was drunk and high, didn’t have control	
Drunk and no control over situation	
Resistance and intoxication	1 (4)
She fought back and I was drunk	
Resistance and environmental risk	1 (4)
Fought back and the surroundings	
Other	3 (13)
The reason was to humiliate the victim. The intent to have sex was not there	
She was dressed in bed	
I was impotent	

## Absence of Risks and Difficulties in Completed Sexual Offences Against Women

Table 4 presents the justifications provided by participants who did not perceive any risk when they committed a sexual offence against a woman. The most common justification given was, by almost half of participants, that in their view, they thought that the sexual assault was consensual (48%). For instance, one participant indicated that: “She was my ex-girlfriend, didn’t notice the fear in her at the time.” Another said: “Thought the victim had consented and was willing.” The next most common justification was intoxication (24%) followed by not thinking about the consequences of the offence (19%). In terms of intoxication, one participant said: “I was enjoying myself too much and was very intoxicated.” Another said: “Under the influence of alcohol and did not consider risk.” Regarding not thinking about the consequences, one participant said: “At the time I did not care about the consequences.” Some other participants reported both consent and intoxication (7%) or the absence of environmental risk (2%).

**Table 4.** Justifications of Perceived Absence of Risks in Completed Sexual Offences Against Women (*n* = 42)

Justifications for absence of risks	Frequency (%)
Thought it was consensual	18 (43)
At the time I thought it was consensual	
She was my wife, and said “yes” at first	
Thought the victim had consented and was willing – She was my ex-girlfriend, didn’t notice the fear in her at the time	
She was my partner	
At the time there was no risk of it being considered a crime (my idea was full consent)	
Cause we were having sex before this it was always consensual	
I knew the person and thought it was consensual	
I thought it was consensual	
I thought it was consensual and that I could trust her	
I thought she gave me consent	
I thought we both wanted it	
She asked me to come to her car for intercourse	
Thought because I paid her double to let me tie her up and try something new it would be okay	
Thought it was sex as part of a normal relationship	
Thought she wanted it	
We had been flirting and thought there was consent	
We were in a relationship and thought it was consensual	
We were in a sexual relationship and had done this type of bondage before	
Intoxication	12 (29)
I was enjoying myself too much and very intoxicated	
I was very drunk	
Off my face with drugs	
Too drunk to care	
Under the influence of alcohol and did not consider risk	
Too drunk to think rationally	
Was heavily intoxicated and blank-minded. Not thinking straight.	
Was not thinking rationally	
I was not thinking rationally as I had been drinking and amphetamines had been placed in my drink	
Very drunk	
Too drunk to care	
I was very drunk	
Did not think of consequences	8 (19)
Anger, revenge, in a rage and didn’t consider risk	
At the time I didn’t care about the consequences	
I was in rage	
Did not think. It just happened.	
Didn’t think	
Didn’t think it was a problem at the time	
I believed at the time it would sooth and fix the fight we had just had	
I didn’t care what I was doing	
Thought it was consensual and intoxication	3 (7)
I was very drunk and she was my girlfriend	
Thought everything was okay, thought she enjoyed it, too drunk to notice or care	
Thought she was okay with it - I was drunk/stoned to notice resistance	
Absence of environmental risk	1 (2)
We were alone as my boy was sleeping	

Table 5 presents the justifications provided by participants who did not perceive any difficulties when committing a sexual offence against a woman. As observed for the absence of risks, the most common justification related to consent (42.1%). Many participants indicated that there were no difficulties related to committing the offence as they thought it was consensual. One participant said: “Because she was already having sex with other people and she was attractive.” Another said: “She agreed to it at the start.” Related to consent, more than a quarter of participants also indicated the absence of resistance from the woman (26.3%). One participant said: “Thought she wanted it, she didn’t resist.” Another said: “Woman did not struggle, too scared.” The other justifications provided by participants related to: (1) Intoxication (8%), (2) Being stronger than the woman (8%), Both intoxication and absence of resistance (5.1%), and (4) Other reasons (10.5%).

**Table 5.** Justifications of Perceived Absence of Difficulties in Completed Sexual Offences Against Women (*n* = 38)

Justifications for absence of difficulties	Frequency (%)
Thought it was consensual	16 (42.1)
I initiated sex as part of the relationship. Thought she wanted to have sex.	
Thought she wanted to have sex	
Because she was already having sex with other people and she was attractive	
She agreed to it at the start	
She initiated it	
She seemed willing to have sexual contact	
She was willing at the time	
At the time I thought she was giving me her consent	
Because it was my partner and make up sex was normal for me	
Cause we were having sex before this it was always consensual	
Prostitute just went along with it	
She was willing at the time and had sex with other boys before	
Thought it was consensual	
Thought the victim had consented and was willing - She was my ex-girlfriend.	
Was my partner and we lived together. She seemed okay with it.	
We were in a relationship and I thought it was mutual	
No perceived resistance	9 (23.7)
Consensual she did not indicate that she did not want to	
I thought she consented. She did not say no	
Thought she wanted it - she didn’t resist	
No resistance	
She was submissive	
Woman did not struggle, too scared	
She was asleep	
She did not resist	
She was drunk and I was sober	
Intoxication	3 (8)
I was heavily intoxicated and did not think about anything	
She was drunk and I was sober	
Victim was asleep, I was high on drugs and had no self-awareness	
Stronger than woman	3 (8)
She tried to fight back but I was stronger	
I was able to overpower her	
She was tied up	
Resistance and intoxication	2 (5.1)
Too drunk to care about her fighting back	
Intoxicated and she did not react	
Other	5 (13.1)
I wanted to gain my life back and the victim was unconscious	
It felt natural at the time	
Thought she was alone. Did not know son was awake	
With her I knew sex would happen. She was the one who got me involved in bondage and I had her in the submissive position	
They were sleeping	

## Discussion

In this study, we examined the perceived risks and difficulties in the context of noncompleted and completed sexual offences against women to help further understand the decision-making process of individuals who commit those crimes to offer complementary insights from a prevention point of view. To do so, we looked at the individuals’ accounts of their offending. First and foremost, the dominating response in regards to high ratings of difficulties in noncompleted offences was woman resistance. Almost 80% of participants indicated that the woman showed resistance during the noncompleted offence. This finding is consistent with the justifications provided by participants who completed an offence. Those participants reported that there were no difficulties related to committing the offence because in their view it was consensual and/or the woman was not resisting during the completed offence. These findings add empirical evidence from the perspective of those who commit crimes to previous literature suggesting that woman resistance, especially what can be considered as forceful resistance, prevents sexual offences (Tark & Kleck, 2004; Ullman, 2022; Ullman & Knight, 1991, 1992). Notably, some participants in completed offence cases described being physically stronger than the woman, implying that attempts at resistance did not prevent the offence. Importantly, while not placing responsibility on women, the evidence suggests that resistance can, in some circumstances, be an effective factor in the prevention of sexual offences. In other words, while findings related to victim resistance could be viewed as victim blaming, they are an important indicator of what may still work when other preventative measures have failed.

Second, participants described intoxication as influencing their perception of risk, with some viewing it as heightening risk and others as diminishing it. Obviously, intoxication decreases cognitive abilities and this has been shown to play a role in sexual offending (e.g., Felson & Burchfield, 2004; Mieczkowski & Beauregard, 2012). One explanation for these findings may relate to the inability of each individual to think through the risk of getting caught when initiating a sexual offence. In some cases, it can be argued that when individuals are intoxicated, they may fail to consider the risk of apprehension and become impaired to the extent of losing control over the situation, thereby increasing their likelihood of being caught and, in some instances, leading to a discontinued offence. This is consistent with previous studies that have found intoxicated individuals less likely to complete a sexual offence against a woman (Brecklin & Ullman, 2001). Conversely, for those who completed an offence, intoxication may have functioned as a facilitating factor, diminishing empathy toward the woman and thereby reducing internal inhibitions that might otherwise have led them to stop. This explanation is also consistent with previous studies indicating that intoxicated individuals were more likely to complete the offence (Brecklin & Ullman, 2002) clearly demonstrating the complex role played by intoxication in different scenarios for different individuals. In this context, intoxication may play a different role based on individual-based differences. For some, intoxication may prevent them from thinking clearly about the risk when initiating an offence, but nonetheless stop when facing other obstacles perhaps because of moral principles surfacing at the time. For others, intoxication may simply be like putting oil on a fire and leave them completely careless about the risk of getting caught and hurting a woman.

The place of intoxication in the current findings clearly suggest that intoxication represents a factor that will shape the decision-making process, which is consistent with the rational choice perspective and SCP in complicated ways. How individuals who commit crime assess the risks and difficulties related to commission of a crime involves different layers of reasoning that will interact and, as such, are difficult to unpack especially when giving consideration to other situational factors and the emotions of individuals involved in a criminal event (see for instance van Gelder et al., 2014). Consistent with SCP, what remains clear is that the judgements made by individuals committing sexual offences regarding risks and difficulties will be shaped by intoxication, and that the impact of intoxication may lead to a different assessment of the situation based on other circumstances and the individual making this assessment.

Third, participants who did not perceive a risk and completed a sexual offence against a woman further indicated, in almost half the cases, that they thought (or assumed) the woman had given her consent. An additional 19% indicated that they did not think or care about the consequences of the offence. These findings may relate to moral inhibitions in many cases or lack thereof and are, therefore, not surprising. It is well demonstrated in the literature that most individuals who commit sexual offences are characterised by various cognitive distortions that help them justify their actions (Marshall et al., 1990). From a SCP point of view, this also brings the importance of measures to remove excuses that can act as precipitators during sexual offences (Wortley, 2001). One way to do this can be to foster explicit and ongoing conversations about consent for both men and women, rather than just assuming it.

Fourth, it is somewhat surprising to observe the difference between the present findings and those obtained in previous studies regarding what is perceived by individuals who commit sexual offences (Beauregard et al., 2012; Beauregard & Leclerc, 2007). In the current study, very few participants referred to environmental conditions when assessing the risks and difficulties of committing an offence. In previous studies conducted by Beauregard and colleagues, perceived risks of apprehension were primarily attributed to the presence of bystanders, the visibility of the location, and whether the victim knew the individual, reflecting important environmental conditions. However, two important points may explain these differences. First, the sample used in those studies consisted of serial sex offences perpetrated by strangers. One possibility is that these individuals are more attuned to planning and thinking about the risks of getting caught as they have committed several offences. Second, in these previous studies, it is also worth noting that participants were asked to provide information on the risks of getting caught at the time when they were planning the offence specifically. In other words, it is possible that they generally consider the risks of getting caught, such as the presence of somebody else witnessing the offence only when contemplating an offence, not during the commission of the offence.

## Considerations for Prevention

Various factors are important to consider from a prevention point of view. The first factor is woman resistance. From a situational prevention perspective, victim resistance in crimes committed against a person relates to making it more difficult for individuals to sexually offend against women (Cornish & Clarke, 2003). It is easy to slip onto this terminology and think victim blaming. Although such findings could be misconstrued as implying victim responsibility, this is not the intention. Rather, the focus is on incorporating evidence-based research, including individuals’ own narratives, to understand situational dynamics. These accounts suggest that when no alternative means of avoidance is available, a woman’s resistance may be the factor most associated with preventing the completion of a sexual offence. In light of these findings, self-defence programs (e.g., Ullman, 2022) and broader initiatives aimed at building resilience and self-efficacy among young women in high school and university settings may represent valuable avenues for prevention, provided they are implemented without implying personal responsibility for victimisation. Further to this, in light of the findings regarding the absence of consent in several cases (as perceived by participants), it would seem that another preventative measure would be to foster explicit and ongoing conversations about consent, rather than just assuming it in the form of education and awareness programs.

The second factor is intoxication, which falls into the strategy of removing excuses used for offending in SCP (Cornish & Clarke, 2003; Wortley, 2001). This strategy involves techniques to better control drugs/alcohol in public facilities, especially, when individuals are showing signs of intoxication. Efforts to reduce offending excuses should also involve increasing men’s awareness of consent and respect for women, whether intoxicated or sober. Interventions such as targeted messaging in alcohol-serving venues may help to trigger reflection and accountability. At the same time, education programs highlighting the impact of intoxication on judgment and vulnerability could further strengthen prevention efforts, provided they do not imply victim responsibility (e.g., Chiu & Leclerc, 2020; Leclerc & Cale, 2015).

The third factor relates to environmental conditions, and more specifically, the role of bystanders. In SCP terms, this refers to extending surveillance and involving place managers into playing a role to prevent crime (Cornish & Clarke, 2003). Even though very few participants in this study reported that the presence of a bystander was considered in their assessment of risks or difficulties, previous studies have shown that individuals who commit sexual offences do consider the presence of bystanders especially before a specific crime is committed (e.g., Beauregard & Leclerc, 2007) and that the presence of a bystander may be one of the strongest predictors to prevent sexual offences (e.g., Chiu & Leclerc, 2019, 2020; Clay-Warner, 2002; Cook et al., 2020), which has been suggested by studies using different data sources (e.g., police reports, participant self-report). The presence of a bystander can be effective in preventing a sexual offence in various ways including: (1) Discouraging an individual from proceeding with the offence during the decision-making process by mere presence or by active supervision, (2) Physically intervening during the offence, or (3) Calling the police. In this sense, measures such as increasing police or security patrol in specific areas, such as nightclubs and restaurants, but also in isolated areas at determined times of the day should be considered in cities where it is not currently the case (see Chiu & Leclerc, 2020).

The current study incorporates unique data on sexual offences against women through the perspective of those who attempted to commit a sexual offence against a woman but were stopped during the incident. Those data focus on justifications provided by individuals who attempted or committed a sexual offence against a woman. Despite this novelty, this study also contains limitations related to asking those individuals to share their insights. Poor memory recall, intoxication at the time of offending, and minimising responsibility due to the desire to be perceived in a positive light by researchers are all factors that could distort the findings to some extent, which is commonly reported in any study examining accounts of those committing crimes. However, despite this possibility, it is important to note that the current findings are quite consistent with previous studies conducted using different methodologies (e.g., police reports) and/or for a different purpose (e.g., treatment) especially regarding the role of victim resistance.

Other limitations include the sample size and the nature of the two groups of individuals. The study would have benefited from more statistical power. The sample size of 145 individuals divided in two groups is relatively conservative and a larger sample size could have permitted conducting more sophisticated analyses with each group of individuals. The history of offending between the two groups of individuals could have an impact on the findings. For instance, the group of individuals who were stopped may have never completed a sexual offence previously. Despite the apparent inequivalence of groups in this regard, it is unclear how a different offending history may impact the findings (see also discussion on self-reported data below). In any matter, it is important to note that only a minority of participants indicated a previous history for sexually offending against a woman (14.1%), which means that this limitation would have a minimum impact on the findings, if any.

Another commonly assumed limitation of self-reported data is that individuals’ descriptions of their offending may differ sharply depending on whether they were ever convicted; for example, unconvicted individuals would be more sophisticated in their criminal behaviour assuming that these same individuals have committed several sexual offences previously to gain offending experience. First, to our knowledge, there is no strong evidence to support this assumption based on current research in the field of sexual offending. Most importantly, it is crucial to remember that individuals convicted of a sexual offence in many cases may have committed multiple other sexual offences for which they were never arrested. In other words, a conviction for a sexual offence does not invalidate the fact that this same individual may have “successfully” committed 10, 20, or 30 other sexual offences for which s/he was not apprehended before. Second, it is worth noting that this study focuses on discontinued offences, which by definition, can only be best documented through self-report data since these incidents are not typically recorded by authorities. Finally, individuals’ accounts of their offending behaviours arguably represent the most informative method to get a closer look at offending behaviours. However, integrating independent sources to bonify these data, such as police or forensic records, or comparing perceptions with objective characteristics of the events, could prove constructive in specific situations.

## Conclusions

Investigating noncompleted sexual offences against women arguably provides additional insights that are not possible to obtain otherwise. Following on the current research stream, examining the configuration of situational factors leading to noncompleted sexual offences against women could prove insightful. These analyses would assist in identifying scenarios most likely to lead to a noncompleted offence, and also whether particular situational factors should be emphasised to inform prevention initiatives (e.g., Chiu & Leclerc, 2020; Hart & Miethe, 2008; Mieczkowski & Beauregard, 2012). Focusing on the relationship between specific variables at the time of the incident, such as victim resistance, intoxication, relationship with the victim, the presence/intervention of a witness, and the crime location for instance, may help identify the optimal combination of situational elements most likely to lead to noncompleted sexual offences against women (see Chiu & Leclerc, 2020; Elffers & Reynald, 2017) and thus best inform crime prevention initiatives.
